# Spatiotemporal Dynamics of Oligofructan Metabolism and Suggested Functions in Developing Cereal Grains

**DOI:** 10.3389/fpls.2015.01245

**Published:** 2016-01-19

**Authors:** Manuela Peukert, Johannes Thiel, Hans-Peter Mock, Doris Marko, Winfriede Weschke, Andrea Matros

**Affiliations:** ^1^Applied Biochemistry Group, Leibniz Institute of Plant Genetics and Crop Plant Research (IPK-Gatersleben)Gatersleben, Germany; ^2^University of CologneCologne, Germany; ^3^Plant Architecture Group, IPK-GaterslebenGatersleben, Germany; ^4^Department of Food Chemistry and Toxicology, University of ViennaVienna, Austria; ^5^Seed Development Group, IPK-GaterslebenGatersleben, Germany

**Keywords:** oligofructan, spatial distribution, grain development, cereals, antioxidant, stress response

## Abstract

Oligofructans represent one of the most important groups of sucrose-derived water–soluble carbohydrates in the plant kingdom. In cereals, oligofructans accumulate in above ground parts of the plants (stems, leaves, seeds) and their biosynthesis leads to the formation of both types of glycosidic linkages [β(2,1); β(2,6)-fructans] or mixed patterns. In recent studies, tissue- and development- specific distribution patterns of the various oligofructan types in cereal grains have been shown, which are possibly related to the different phases of grain development, such as cellular differentiation of grain tissues and storage product accumulation. Here, we summarize the current knowledge about oligofructan biosynthesis and accumulation kinetics in cereal grains. We focus on the spatiotemporal dynamics and regulation of oligofructan biosynthesis and accumulation in developing barley grains (deduced from a combination of metabolite, transcript and proteome analyses). Finally, putative physiological functions of oligofructans in developing grains are discussed.

## Introduction

**FIGURE 1:**
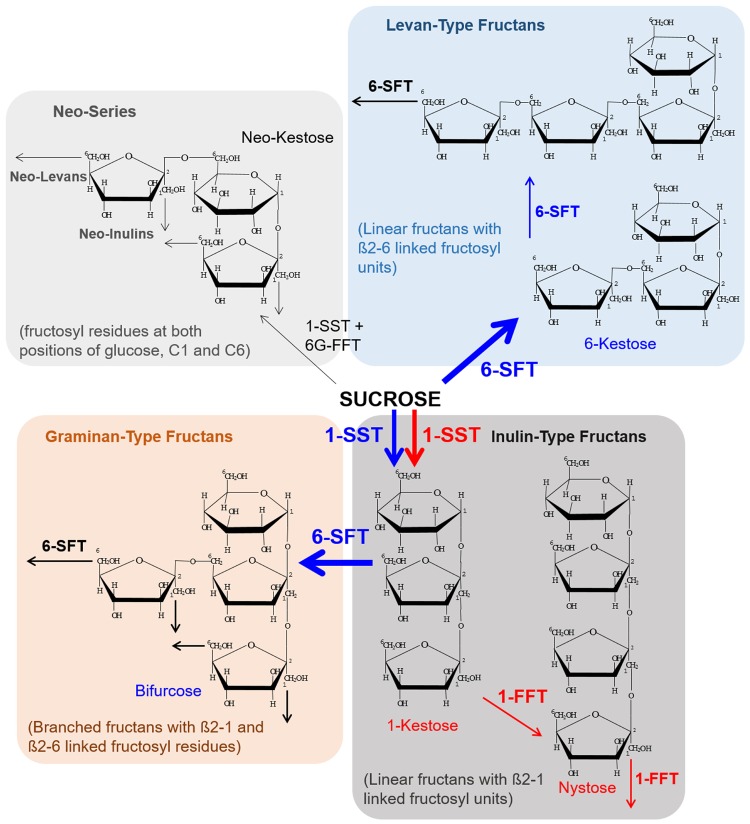
**Representation of the various fructan-types and suggested biosynthesis routes in developing barley grain tissues.** A spatiotemporally specific coordinated biosynthesis of oligofructans has been observed for barley grains. The pathway in blue illustrates the major route of biosynthesis during the prestorage phase and red indicates the major route during the storage phase. During the prestorage phase a high transcript level for 1-SST and 6-SFT was observed for the endosperm leading to an accumulation of 6-kestose and bifurcose (indicated in blue). With transition to the storage phase a transcriptional switch was observed resulting in high transcript levels of 1-SST in the nucellar projection (NP). 1-FFT was found to be exclusively expressed in the NP during the storage phase. Induction of the inulin-type oligofructan biosynthesis pathway led to high amounts of 1-kestose and nystose accumulated in the endosperm cavity (indicated in red). Oligofructans of the neo-series were not detected in developing barley grains. Abbreviations are: 1-FFT, fructan:fructan 1-fructosyltransferase; 1-SST, sucrose:sucrose 1-fructosyl-transferase; 6-SFT, sucrose:fructan 6-fructosyltransferase; 6G-FFT, fructan:fructan 6G-fructosyltransferase.

Starch, fructans and β(1,3; 1,4)-glucans represent the major plant reserve carbohydrates (Vijn and Smeekens, 1999; Burton and Fincher, 2009). Among them, fructans form a complex carbohydrate class which is produced in only about 15% of higher plants, including cereals, vegetables, ornamentals, and forage grasses (Hendry and Wallace, 1993; Vijn and Smeekens, 1999; Cairns et al., 2000; Van Den Ende et al., 2004). Fructan biosynthesis evolved polyphyletically. This is reflected in the diversity of fructan accumulation among dicotyledonous and monocotyledonous plant species. While dicots accumulate fructans mainly in their below-ground reserve organs (roots, tubers), monocots typically store fructans in above-ground parts of the plants (stems, leaves, seeds). Fructans consist of repeating fructose residues linked to a sucrose unit. They can form polymers [with a degree of polymerization (DP) equal or greater than 10] or oligomers with a small number of monomers (with DP 3–9), also referred to as oligofructans, fructooligosaccharides (FOS) or oligofructose. In the following, the term fructans is used, when no differentiation has been made between FOS or fructan polymers. In the majority of available literature fructans are discussed in general manner without differentiation of the DP. Since fructans came into a more widespread focus of interest, recently more attention is paid to discuss the role of fructans dependent on their level of DP. Different classes of fructans are distinguished according to the position of the sucrose moiety, the kind of linkage between the fructose residues [β(2,1), inulin; β(2,6), levan or containing both β(2,1) and β(2,6)-D-fructosyl units, graminan-type] and the chain lengths (John, 1992; Pollock and Cairns, 1999; Cochrane, 2000). Fructan biosynthesis includes the activity of various fructosyltransferases (FTs) that have been described for several plant species (Vijn and Smeekens, 1999), and have been illustrated in **Figure 1**. All aforementioned types of fructans are known to occur in *Poaceae* (Bancal et al., 1991; Carpita et al., 1991; Pollock and Cairns, 1991; Fretzdorff and Welge, 2003). A differentiation in botanical subgroups according to predominant fructan structures showed that *Triticum*, *Secale*, and *Hordeum* mainly contain fructans of the branched-type (graminan) whereas the tribe of *Poodae* is predominantly characterized by levan-type linkages (Bonnett et al., 1997; Huynh et al., 2008). However, structural variations between different plant organs are not excluded.

Fructans in cereals accumulate in stems and leaves (Wagner and Wiemken, 1987; Van Den Ende et al., 2003), as well as in grains (Biesiekierski et al., 2011; Verspreet et al., 2013a). Cereal grains are the worldwide most important energy sources of human and animal nutrition, comprising about 50% of all food for human consumption (Loftas et al., 1995). Most important cereals are maize, rice, wheat, barley and sorghum, with barley on the fourth place of the cereal world production as reported in the Faostat 2013 statistics^1^. The usage of cereal grains for food and feed or further processing (e.g., biofuels production) is determined by the structural and nutritional composition of the mature grain. The major components are starch, fiber (non-starch polysaccharides including fructans), proteins, soluble sugars, lipids, and minerals (Byung-Kee et al., 2011). Even though cereal grain composition is of high scientific and industrial interest, the complex physiological changes occurring in developing grains are far from being fully understood. Especially, the fate of oligofructans in cereal grains and their particular functions during grain development have been poorly investigated.

An increasing interest in oligofructan biosynthesis and its physiological functions can be monitored over the last decade. About half of the published reports on ‘*fructan and plants*’ (246) were related to cereals, mostly wheat (128) and barley (43); but also rye (16), oat (12) and maize (10). Among them, a relevant part of reports was related to fructan metabolism in grains (58)^2^. As in other research fields of modern plant biology, increased attention has been paid to the regulation of molecular processes down to the tissue and cellular level. Recent results obtained from tissue-specific studies of oligofructan metabolism in barley grains are discussed here.

## Oligofructans in Cereal Grains

### Oligofructan Amounts in Mature Grains Vary Between Cereal Species and Differ in Their Degree of Polymerization (DP)

The presence of fructose polymers (firstly named ‘*fructosans*’) in cereal grains has firstly been recognized in the late 19th century, and from the 1940s new interest in cereal grain sugar metabolism was recorded (Archbold, 1940; MacLeod, 1952). Meanwhile, cereal oligofructans have become an object of scientific interest, particularly because of their role in grain development and as a dietary fiber for human nutrition. However, grain fructan concentrations have only been rarely resolved on their DP level. Usually, determination of fructan concentration was based on acid hydrolysis or enzymatic digestion and further quantification of fructose in relation to glucose (Verspreet et al., 2015). From analyses differentiating the polymerization status it was concluded that the major fraction of grain fructans belongs to oligofructans (Henry and Saini, 1989; Verspreet et al., 2015). The total concentrations of fructans in mature grains are highly variable, depending on the cereal species and the respective variety. Rice and maize are generally designated as non-fructan plants due to their very low or even non-detectable amounts of fructans (Pollock and Cairns, 1991; Stoop et al., 2007; Kawakami et al., 2008; Biesiekierski et al., 2011). Besides, oat contains only traces to 0.2 mg/g dry mass (DM; Aman, 1987; Henry and Saini, 1989) whereas in rye the highest fructan concentrations have been detected, ranging from 1.7 to 6.6% of DM (Henry and Saini, 1989; Karppinen et al., 2003; Fretzdorff and Welge, 2003). Fructan values for barley vary between traces to 1% of DM (MacLeod, 1952; Aman et al., 1985; Henry and Saini, 1989) to 4.2% of DM (Nemeth et al., 2014). Similar fructan concentrations have been found in wheat (1.4–2.3% of DM; Fretzdorff and Welge, 2003; Huynh et al., 2008), Einkorn (1.6–2.2% of DM; Brandolini et al., 2011), triticale (1.8% of DM), durum wheat (1.6% of DM), and spelt (1.1% of DM; Fretzdorff and Welge, 2003). The degree of polymerisation (DP) of oligofructans in barley changes with increasing oligofructan concentrations (Nemeth et al., 2014). In 1989, Henry and Saini revealed differing amounts for oligofructans with 2.6 (DP 3), 2 (DP 4), 0.3 (DP 5), and 2.33 mg/g DM (DP > 5) in mature barley grains, which was confirmed by results from Xue et al. (2011). The interested reader is also referred to a recent review (Verspreet et al., 2015).

### Genotypic and Environmental Factors Determine Fructan Contents in Cereal Grains

Recent results on the health promoting implications of plant prebiotics, such as fructans, have promoted the screening of germplasm collections and biotechnological approaches to increase the content of oligofructans in classical non-fructan cereals, such as maize (Dwivedi et al., 2014). Among cereal crops, largest genetic variations have been reported for barley and wheat, and a number of quantitative trait loci (QTL) have been identified for high fructan content in wheat already (Dwivedi et al., 2014). Besides genotypic variation, environmental factors affect cereal grain fructan content. For example, grain fructan concentrations from field-grown barley and wheat lines were nearly duplicated when compared to concentrations obtained from greenhouse trials (Huynh et al., 2008). Results from field trials at five different locations indicated that environmental factors have a strong impact on final fructan concentration in wheat and rye (Fretzdorff and Welge, 2003). Contrary, Brandolini et al. (2011) detected no effects regarding the location, but strong impact of the year of cultivation. Results from Karppinen et al. (2003) also revealed year-dependent changes in rye in different varieties. In summary, both the genetic variation and the genotype × environment interactions will provide the basis for further improvement of cereal grain quality with respect to nutrition and health promotion. In particular, the availability of germplasm collections with large genetic variation for oligofructan content will enable more detailed studies on oligofructan function in the near future.

### Biosynthesis of Oligofructans Follows Grain Development with Highest Amounts at the End of the Prestorage Phase

The oligofructan composition of grains is established during development. Before starch accumulation is initiated, a considerable amount of carbon is directed toward the building of low molecular weight oligofructans. Their concentration peaks at the end of the prestorage phase (7–10 days after pollination, DAP) and decreases during storage product accumulation. Fructan concentrations up to 35% of DM in wheat and durum wheat, and 39% of DM in barley have been reported (De Gara et al., 2003; Verspreet et al., 2013b). In triticale and rye grains the fructan concentration follows the same trend during development with a decrease from 16.7 to 6.2% in rye and 23.7 to 3.4% in triticale between 9 and 28 DAP (Nardi et al., 2003). Furthermore, the average DP changed from DP 7–8 to DP 4–5 (Verspreet et al., 2013a; Cimini et al., 2015). Results from correlating enzyme assays in wheat (Verspreet et al., 2013a) complement the metabolite variations pointing toward strong temporal coordination of oligofructan metabolism. Considering the mixture of tissues in developing grains, a differentiation with regard to tissue-specific metabolic features is neccessary to finally draw conclusions about putative functions.

## Spatiotemporal Dynamics of Oligofructan Metabolism in Cereal Grains

### Oligofructan Metabolism in Wheat Grains

#### Tissue Specific Oligofructan Distribution

In order to elucidate the composition of the apoplastic sap in the endosperm cavity and its relevance for grain filling, Lim (1988) firstly described an accumulation of ‘*fructosans*’ in the apoplastic space. He found that fructans encompassed 88% of the total sugar weight with a concentration range between 54 and 129 mg/ml. During early development of wheat grains, highly intensive oligofructan partitioning to the outer pericarp has been observed (Housley and Pollock, 1993; Schnyder et al., 1993), making up 75% of all water soluble carbohydrates (WSC) in this tissue region at five DAP. Later, when the pericarp disintegrates also oligofructan levels strongly decrease in the pericarp (Schnyder et al., 1993). The observed differences of fructan content in particular grain parts are kept until maturity in wheat and are likely conserved among most cereal species. The bran of mature wheat and rye grains contains fructan amounts of 3.7% (wheat) and 6.6% (rye) compared to flour with 1.5% (wheat) and 4.5% (rye) (Karppinen et al., 2003; Haskå et al., 2008). These variances in oligofructan accumulation of mature grains are particularly important with respect to food processing and improvement of the nutritional quality of cereal products. As oligofructans and inulin are the best-characterized plant prebiotics (Dwivedi et al., 2014), whole grain products provide an important strategy in increasing the levels of prebiotics in staple food crops, and thus to enhance nutrition and health.

#### Temporal Patterns of Transcripts of Fructan Metabolism Genes

In wheat grains, fructan metabolism has been studied intensively from anthesis until maturation by analyzing fructan concentrations and enzyme activities of fructan metabolism (Verspreet et al., 2013a). Only at earlier stages of grain development (until 14 DAP), notable accumulation of fructans and corresponding enzyme activities were detected. Coincidently, the total activity of FEH enzymes degrading fructans peaks at later stages between 20 and 28 DAP, when sucrose levels are decreased. This is in accordance to results from the forage grass *Lolium perenne*, for which strong inhibition of FEH activity by high levels of sucrose have been reported (Lothier et al., 2014). Similar results about changing sugar concentrations and oligofructan metabolism during grain development have been reported by Cimini et al. (2015) for durum wheat. During early development (seven DAP) when cellularization is finished and the differentiation of the starchy endosperm is initiated, high hexose levels are accompanied by high oligofructan concentrations, particularly of oligofructans with a higher DP. Additionally, the authors found positive correlations between temporal oligofructan accumulation patterns and the expression levels of biosynthesis genes (6-SFT, 1-FFT, 1-SST) but also of genes encoding degrading enzymes by performing semi-quantitative RT-PCR analysis. The results indicate that oligofructan metabolism is tightly regulated in a temporal manner of wheat grain development and point toward a possible correlation of oligofructan metabolism to grain developmental processes.

### Oligofructan Metabolism in the Developing Barley Grain

Most of the knowledge about oligofructan metabolism in cereal grains is gained from analyses of the complete grain, which neglects potential differences between grain compartments and/or distinct cell types. To overcome this limitation in spatial resolution, tissue-specific transcript, metabolite and proteome analyses have been performed in developing barley grains from the prestorage/differentiation to the storage phase (Peukert et al., 2014).

#### Spatiotemporal Patterns of Oligofructan Distribution

**FIGURE 2:**
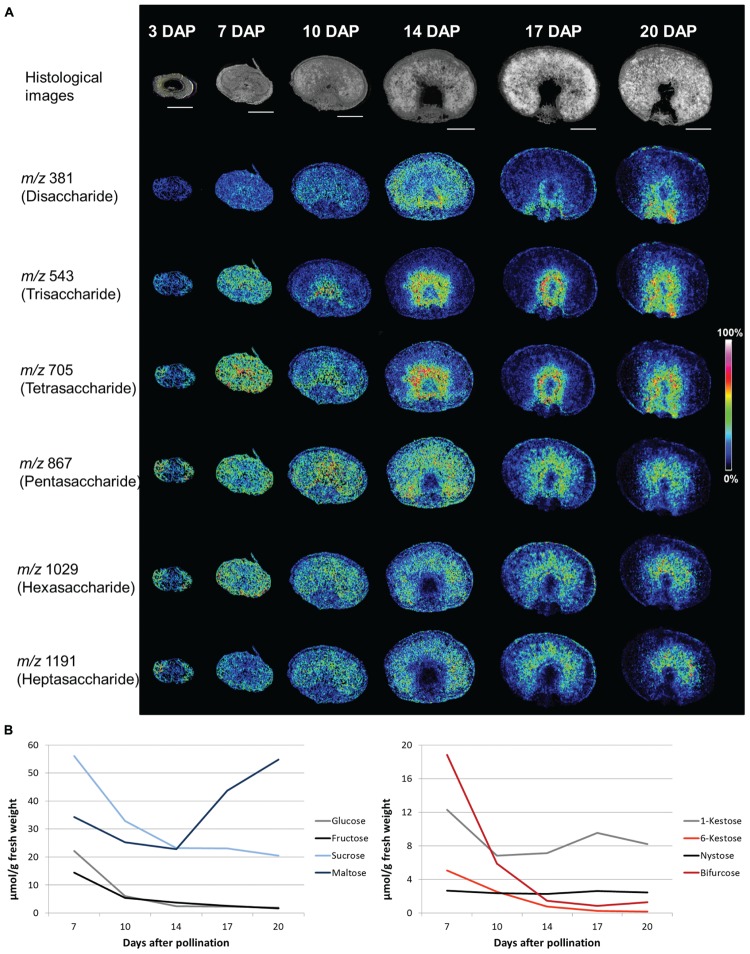
**Sugar contents in developing barley grains.** **(A)** Accumulation patterns of oligosaccharides during barley grain development as observed by MALDI MSI. The top panel shows histological images illustrating the various developmental stages which were analyzed. The following panels show the ion intensity maps of the disaccharide (*m/z* of 381), the trisaccharide (*m/z* 543), the tetrasaccharides (*m/z* 705), the pentasaccharide (*m/z* 867), the hexasaccharide (*m/z* 1029), and the heptasaccharide (*m/z* 1191). Specific accumulation of the tri- and tetrasaccharides in and around the nascent endosperm cavity was observed from 10 DAP onwards. The penta-, hexa, and heptasaccharides, which accumulated in the pericarp during the prestorage phase (three DAP), moved to the endosperm at the beginning of the storage phase (from 10 DAP onwards). Bars = 1 mm. The images represent a reprint of Figure 2 and Supplemental Figure S2 from Peukert et al. (2014). **(B)** Quantities of sugars in total grains (μmol/g fresh weight). Hexoses (glucose and fructose) and sucrose decreased from 7 DAP until 20 DAP whereas maltose strongly increased (left diagram). For the oligofructans 6-kestose and bifurcose [both containing β(2,6)-linkages] a steep decline during the storage phase was observed. For the inulin-type oligofructans, 1-kestose and nystose, less pronounced changes were observed in the whole grain. Sugar quantities are graphically presented according to the data from Supplemental Table S1 of Peukert et al. (2014) (www.plantcell.org). Copyright American Society of Plant Biologists.

Making advantage of recent developments in analytical technologies detailed studies of sugar distribution in particular tissues or even cells became feasible. In plant biology, mass spectrometry imaging (MSI) based on matrix-assisted laser-desorption ionization (MALDI) has been established to elucidate the spatial distribution of certain classes of metabolites (e.g., of lipids and sugars), peptides or small proteins (Peukert et al., 2012; Matros and Mock, 2013). Application of MSI technology enabled the visualization of spatiotemporal patterns of oligosaccharide distribution during barley grain development. In the young grain (three DAP), most of the oligosaccharides (DP 4–7) accumulate in the pericarp. When endosperm tissues are differentiated oligosaccharides of DP 2–7 are uniformly distributed. At the early storage phase (10 DAP), increased amounts of tri- and tetrasaccharides have been observed in the cells surrounding the nascent endosperm cavity, which becomes more prominent during the storage phase (14–20 DAP). Those tri- and tetrasaccharides were identified as the inulin-type oligofructans 1-kestose (DP 3) and nystose (DP 4, Peukert et al., 2014). For the oligofructans 6-kestose (DP 3, levan-type) and bifurcose (DP 4, graminan-type) highest concentrations have been found at seven DAP in both dissected transfer region and remaining grain. In comparison, the inulin-type oligofructans 1-kestose and nystose are much lower concentrated at seven DAP and their concentrations in the dissected transfer region are similar to those obtained for the remaining grain. With transition to the storage phase 6-kestose and bifurcose decline (between 7 and 10 DAP) whereas oligofructans of the inulin-type accumulate in the transfer region (**Figure 2**). This pattern of localization persists until the end of the grain filling period (20 DAP). These tissue specificities would have been neglected in whole grain samples (**Figure 2B**), where the particular amounts of 1-kestose, 6-kestose, bifurcose, and nystose have been found to decrease between 7 and 20 DAP. From the distinct spatiotemporal distribution patterns of 1-kestose and nystose it has been concluded that accumulation in transport active tissues during the storage phase might be related to protective functions of inulin-type oligofructans by maintaining high import rates into the endosperm (Peukert et al., 2014). Assuming similar distribution patterns for other cereals, functional studies will help to elucidate generalized functionalities of particular oligofructan-types in plants in the near future.

#### Spatiotemporal Transcript Patterns of Genes Related to Fructan Metabolism

Transcript profiling of genes encoding fructan metabolic enzymes has been reported from laser-captured microdissected grain tissues playing a pivotal role for grain filling, namely the nucellar projection (NP) and the endosperm transfer cells (ETC), as well as the endosperm (Peukert et al., 2014). 6-SFT and 1-SST depict a high expression predominantly in the early endosperm (5–7 DAP) whereas with the beginning of the grain filling period (10 DAP) transcript levels decreased dramatically. Concerted transcriptional activities of 1-SST and 6-SFT correspond to the high abundance of bifurcose and 6-kestose, graminan- and levan-type oligofructans, in the young grain (Peukert et al., 2014). Together, this indicates a *de novo* biosynthesis of the branched oligofructans from sucrose in the early endosperm in a combined operating mode of both enzymes, which has also been proposed for wheat grains (Verspreet et al., 2013a; Cimini et al., 2015). Between 7 and 10 DAP, 1-SST and 1-FFT are co-expressed in the NP, whereas 1-FFT was non-detectable in the analyzed endosperm tissues. Gene expression patterns of 1-SST and 1-FFT tightly correlate to the start of 1-kestose and nystose accumulation in the transfer region/cavity sap (**Figure 2**) during the onset of grain filling. The results additionally point to the universal role of 1-SST in oligofructan metabolism with an interdependency either with 6-SFT in the early endosperm or with 1-FFT in the upper subdomain of NP (two enzymes with different features concerning the degree of oligofructan branching). Among the fructan degrading enzymes, only 1-FEH showed a relevant expression in barley grain tissues. 1-FEH expression overlapped with 1-SST and 1-FFT expression in the upper part of the NP at 10 DAP, exactly the region where 1-kestose and nystose are deemed to be synthesized (Peukert et al., 2014). No transcriptional activities in barley grain tissues (also in the endosperm) were yet reported for 6-FEH potentially involved in 6-kestose degradation. Generally, oligofructans are depolymerized by 6-FEH and 1-FEH enzymes following source–sink modifications such as wounding (e.g., for forage grass species) or grain filling (Schnyder, 1993; Lothier et al., 2014). More recently, the contribution to abiotic stress tolerance probably by regulating the cellular osmotic status and/or stabilization of membranes during frost or drought has been proposed for oligofructans (Hincha et al., 2007; Livingston et al., 2009). These proposed functions presume a concerted action of oligofructan biosynthesis and degrading enzymes. In this respect, spatiotemporal transcript abundancies as shown for barley grains (Peukert et al., 2014) or *L. perenne* leaf sections (Lothier et al., 2014), point to functional distinctions in the various tissues.

#### Disturbed Sucrose Import into the Barley Endosperm Alters Oligofructan Metabolism

To get information about the cross-talk of sugar import into the endosperm and oligofructan metabolism, the shrunken-endosperm mutant *seg8* (Felker et al., 1985; Röder et al., 2006) was used as a model for sucrose re-allocation and depletion (Melkus et al., 2011). *Seg8* shows an impaired differentiation in the transfer-related grain tissues, NP, and ETC, which is probably induced by altered gibberellic acid (GA) and abscisic acid (ABA) balances (Weier et al., 2014). Impaired differentiation compromises transfer of assimilates, causes strongly reduced endosperm filling and thereby, reduces grain weight by up to 70%. MALDI-MSI of wild type (WT) ‘Bowman’ and mutant grains showed altered oligosaccharide distribution patterns at the early storage phase (Peukert et al., 2014). The trisaccharides (predominantly 1-kestose) and tetrasaccharides (predominantly nystose) usually accumulating in the WT tissues around the endosperm cavity are barely detectable in the *seg8*-grains, indicating that the characteristic distribution pattern of inulin-type oligofructans is not established in the mutant. Similarly, an accumulation of oligosaccharides with DP 5 and 6 in the endosperm at the beginning of the storage phase has been observed for the WT, while being hardly detectable in *seg8*-grains. This reveals that biosynthesis of higher polymeric carbohydrates might be disturbed. Consistently, fructan synthesis genes (1-SST, 6-SFT, and 1-FFT) showed diminished expression in the early endosperm (5 DAP) and in the NP at the onset of grain filling (10 DAP) in *seg8* relative to the WT. Similarly, no transcripts for the degrading enzyme 1-FEH could be detected in the NP at 7 and 10 DAP and thereby, showing a tissue-specific co-suppression in the mutant. The co-expression of 1-FFT and 1-FEH in the same cellular region (NP) was also shown in the WT background ‘Bowman’ and in the genotype ‘Barke’ implying a general stimulated metabolism of nystose by simultaneous gene expression of biosynthetic and degrading enzymes (Peukert et al., 2014). These results indicate that disturbances of proper differentiation of the transfer tissues NP and ETC, and thus decreased sucrose flux toward the endosperm in *seg8*, strongly impact fructan gene expression and biosynthesis in both, maternal NP and developing endosperm. The transcription of fructan biosynthesis genes might be induced by high sucrose levels and thereby, a regulatory role especially for 6-SFT in maintaining the hexoses/sugar oligomers balances can be assumed (Martinez-Noel et al., 2009).

### Signals Responsible for the Initiation of Oligofructan Biosynthesis in the Developing Grain

Barley genes encoding 6-SFT and 1-SST have been cloned previously (Sprenger et al., 1995; Nagaraj et al., 2004). However, the inductive signals responsible for the initiation of fructan biosynthesis in the developing grain, particularly in its spatial and temporal context, remained largely unknown. It has been shown by promoter-GUS fusions that 6-SFT is transcriptionally induced in barley leaves by sucrose and light (Nagaraj et al., 2001). 1-SST transcripts are rapidly induced by exposure to light (significant increase within 1 h). Under the same conditions, 6-SFT transcript levels remained low during the first 2 h of illumination and increased thereafter (Nagaraj et al., 2004). In contrast to 6-SFT, 1-SST expression immediately decreased after transfer to darkness (<30 min); this is clearly before the sucrose pool derived from photosynthetic assimilates gets depleted. This implies that 1-SST might be a kind of sensor for changing environmental conditions and thereby, for changing sugar supply. According to the 1-SST/6-SFT model for biosynthesis of graminan-type fructans (Wiemken et al., 1995), 1-SST can be regarded as a ‘pacemaker’ enzyme for fructan biosynthesis. This seems to be reasonable due to the fact that most of the fructan types are derived from 1-kestose which is produced by 1-SST using sucrose as an acceptor (see also **Figure 1**).

#### The Crosstalk Between Sucrose Signaling and Hormones

Sucrose was indicated as a major trigger in transcriptional activation of genes encoding fructan biosynthetic enzymes (Wiemken et al., 1995; Wang et al., 2000; Nagaraj et al., 2001, 2004). Sucrose induction of gene expression is likely mediated by transcription factors and hormonal influences. A summary of hormonal influences on oligofructan metabolism is given by Valluru (2015) integrating latest results about cross-talk of hormones and fructan metabolic enzymes. One prominent example is the sucrose-inducible TaMYB13 transcription factor, which has been shown to directly induce the promoter activities of wheat 1-SST, 6-SFT, and 1-FFT genes by transactivation assays (Xue et al., 2011; Kooiker et al., 2013). Similarly, TaMYB13 DNA-binding sites have also been found in the promoter region of barley 1-SST, 6-SFT, and 1-FFT genes, of which 1-SST and 6-SFT are co-expressed in barley seedlings and complete caryopsis (Huynh et al., 2012). The cross-talk between sugar signaling and ABA, especially the promotion of starch biosynthesis and carbohydrate storage is well known (Rook et al., 2001; Arroyo et al., 2003; Osuna et al., 2007; Weichert et al., 2010). Additionally, the promoter of the barley 6-SFT gene contains several *cis*-regulatory elements associated to ABA signaling (Nagaraj et al., 2004). Enhanced expression of fructan biosynthetic genes by exogenous ABA application was shown in agave (Suarez-Gonzalez et al., 2014) and ABA-dependent regulation was concluded for the 1-FEH gene from chicory (Michiels et al., 2004). In cereal grains, ABA was found to accumulate in starchy endosperm, aleurone cells, embryo and testa (Brenner and Cheikh, 1995). Transcriptome analyses of the developing rice endosperm (Xue et al., 2012) and the proliferating *Arabidopsis* endosperm at the syncytial stage (Day et al., 2008) revealed a pronounced expression of ABA biosynthesis and signaling genes during early endosperm differentiation. Together results hint at an important role of ABA in endosperm differentiation and correspond to the results for the barley *seg8* mutant (Weier et al., 2014).

Altered ABA levels and signaling pathways have been attributed to defects in cellularization of ETCs and the middle part of the endosperm (Sreenivasulu et al., 2010). Transcripts of 6-SFT were predominantly found in the young barley endosperm at five and seven DAP and thereby, indicating ABA influences on transcriptional activity of 6-SFT. This assumption is supported by the known presence of *cis*-regulatory elements linked to ABA signaling in the 6-SFT promoter (Nagaraj et al., 2004).

The repression of 6-SFT in the *seg8* endosperm at five DAP might be correlated to the strong reduction of ABA concentrations in the mutant during early development (Sreenivasulu et al., 2010; Weier et al., 2014). 1-SST is concertedly down regulated in the *seg8* endosperm and co-expressed with 6-SFT in the WT ‘Bowman’ endosperm at five and seven DAP (Peukert et al., 2014), which indicates common modes of transcriptional regulation with a major role for ABA. 1-SST is also expressed in the upper part of the NP at the time when grain filling starts. Spatially, 1-SST expression overlaps with that of 1-FFT and 1-FEH, which are expressed specifically in the meristematic region of the NP. Elongation of NP cells in barley is probably regulated by GA (Thiel et al., 2008) and GA/ABA balances determine the differentiation gradient within the NP (Weier et al., 2014). In *seg8* grains, the transient increase in concentrations of bioactive GAs between 7 and 11 DAP was delayed or less pronounced probably associated to the strong suppression of HvGA20ox1 gene activity in *seg8* NP (Weier et al., 2014). Subsequently, reduced GA levels and GA signal transduction in the mutant NP might be the reason for repression of 1-FFT and 1-FEH transcript levels resulting in the disturbance of the characteristic distribution pattern of inulin-type oligofructans in *seg8* at the beginning of the grain filling period. Collectively, the results hint at a cross-talk of sugar/sucrose signaling and hormones in transcriptional regulation of oligofructan metabolism and consequently, in the establishment of development- and tissue-specific oligofructan accumulation patterns.

## Functions of Fructans and Fructooligosaccharides in Cereal Grains

**FIGURE 3:**
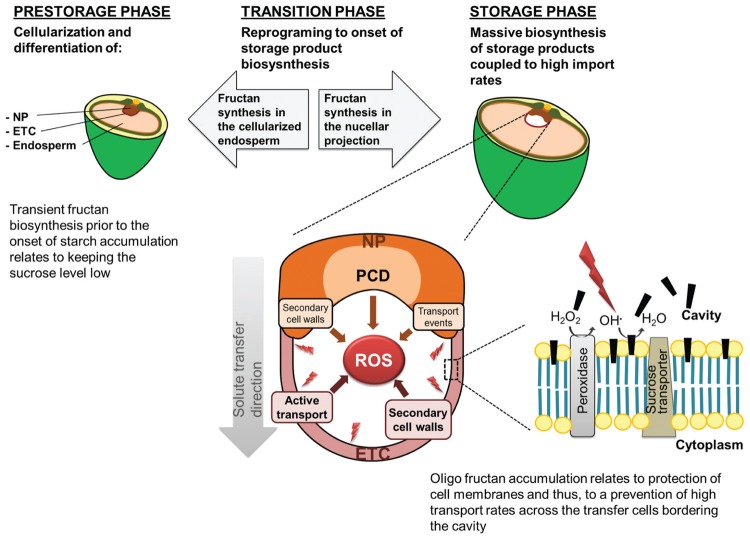
**The proposed functions of oligofructans during barley grain development.** Conversion of sucrose into oligofructans during the prestorage phase is supposed to maintain a high glucose to sucrose ratio in the developing endosperm and thus preventing premature differentiation into storage cells. During the storage phase inulin-type oligofructans accumulate in the transport tissues presumably protecting transport active cells from ROS-inflicted oxidative damage by sequestration into their plasma membranes. The black bars indicate the hypothesized insertion of oligofructans between the head groups of membrane phospholipids.

Functions of fructans have mostly been related to vegetative tissues, where they are implicated in carbohydrate partitioning as an alternative to starch (Albrecht et al., 1997; Cairns, 2003), maintenance of source-sink gradients (Pollock et al., 2003), and short/long-term storage (Nelson and Spollen, 1987; Schnyder, 1993; Housley, 2000). Also protective functions against various abiotic stresses have been proposed for distinct oligofructans (Livingston and Henson, 1998; Valluru et al., 2008; Livingston et al., 2009; Van Den Ende and Valluru, 2009; Keunen et al., 2013). In cereal grains, the spatiotemporal dynamics of oligofructan distribution patterns during development points toward versatile functions (see also **Figure 3**).

### During Early Grain Development, Oligofructans are Potentially Involved in Balancing the Sucrose Level

The presence of fructans in grains has been associated with osmoregulation during cell expansion and growth as well as sucrose phloem unloading (Schnyder et al., 1993; Pollock and Cairns, 1999; De Gara et al., 2003). During the prestorage phase, most assimilates are delivered to the pericarp, which undergoes rapid growth immediately after anthesis. Later, when the endosperm undergoes cell expansion (due to high net water uptake and formation of vacuolated cells), assimilates are supplied to the endosperm and oligofructan concentration increases. Oligofructan synthesis in the differentiating endosperm is suggested to reduce the osmotic consequences of excess amounts of sucrose by sequestering the surplus from ongoing sucrose import. At the same time, biosynthesis of oligofructans is supposed to maintain the gradient of sucrose between the vascular bundle and the sink tissues (Pollock and Cairns, 1991). The tissue-specific analysis of the dynamics of oligofructan metabolism in developing barley grains revealed the particular biosynthesis of levan- and graminan-type oligofructans in the cellularized endosperm, with a peak at seven DAP, prior to the beginning of starch synthesis (Peukert et al., 2014). Probably, sucrose entering the endosperm -before starch biosynthesis starts- is processed into oligofructans. Besides the formerly assumed function in osmoregulation, transient carbon partitioning by oligofructan biosynthesis might reduce the level of sucrose in order to prevent precocious accumulation processes in the endosperm cells. By depletion of sucrose used for oligofructan biosynthesis a high glucose/sucrose ratio is maintained, which was found to be characteristic for cellularization and cell expansion in the cereal prestorage endosperm (Weber et al., 1996a; Weschke et al., 2003; Koch, 2004; Ishimaru et al., 2005). Recently, cell wall invertases, which hydrolyze sucrose into glucose and fructose, have been shown to be expressed only in the early endosperm undergoing nuclear division in *Arabidopsis* and cotton (Wang and Ruan, 2012). Furthermore, glucose signaling has been shown to regulate filial cell division (Weber et al., 2005) and to modulate the expression of some regulatory genes (Wang et al., 2014). In addition, the energetic costs for the transient processing of sucrose into oligofructans are low as both, synthesis and breakdown, are rapidly and easily accomplished (Van Den Ende, 2013). With transition to the storage phase, oligofructan biosynthesis declines in the endosperm and the glucose/sucrose ratio decreases. The level of sucrose has been reported to correlate with the onset of storage product synthesis in legumes (Weber et al., 1996b, 1998), barley (Weschke et al., 2000), wheat (Verspreet et al., 2013b), and cassava tubers (Baguma et al., 2008). Consistently, the transient expression of SUSIBA2, a regulatory transcription factor of starch biosynthesis, correlates with high endogenous sucrose levels in the barley endosperm at 12 DAP (Sun et al., 2003).

The suggested physiological functions of oligofructan biosynthesis during early grain development still remain to be proven. It can be concluded that fructan biosynthesis during the prestorage phase plays a significant role for cellularization processes by affecting the level of sucrose. A reduction of the sucrose level might have an impact on the osmotic status of the cells, on sink stimulation or it implies an inhibition of a signal that would lead to a precocious differentiation into storage cells. For detailed studies it would be necessary to create barley lines with a reduced or inhibited tissue-specific biosynthesis of oligofructans. In this direction, RNAi-lines with either inducible promoters or tissue-specific promoters (e.g., pericarp-specific or endosperm-specific for knock-down of 6-SFT and 1-SST) would be highly valuable. Furthermore, much more information is needed about transcriptional activation of fructan biosynthesis genes. The obtained tissue- and temporal-specific patterns of fructan accumulation point toward a tight development-dependent regulation, so that disturbances of the early grain fructan levels are speculated to result in disturbed grain development.

### During the Storage Phase, Small Inulin-Type Oligofructans are Potentially Involved in the Protection of Transfer Tissues from Oxidative Damage

During the prestorage phase the young endosperm is characterized by high mitotic activity and cell expansion in relation to the net uptake of water (Schnyder et al., 1993). The highly vacuolated barley endosperm cells accumulate levan- and graminan-type oligofructans. As dividing and metabolic active cells are susceptible to reactive oxygen species (ROS), a role for oligofructans in ROS detoxification in the endosperm has also been supposed (Verspreet et al., 2013b). At the onset of the storage phase, ETCs and the NP are fully differentiated in the barley grain. ETC and NP cells depict characteristic transfer cell morphology and the endosperm cavity begins to form (Thiel et al., 2012). At the same time inulin-type oligofructans start to accumulate in and around the nascent endosperm cavity. An orchestrated action of transporter proteins, H^+^-ATPases and cation channels is necessary to supply the expanding endosperm with assimilates and nutrients (Weschke et al., 2000; Offler et al., 2003). The generation of ROS might be a side-product of these transport activities (Luthje et al., 2013). Furthermore, the process of programmed cell death (PCD) in the central parts of the NP probably contributes to ROS generation. Also, cell wall peroxidases are involved in ^•^OH-induced cell-wall loosening (Chen and Schopfer, 1999; Heyno et al., 2011), necessary for secondary cell wall ingrowths production (Cosgrove, 2005; Kim and Triplett, 2008). Based on these data, it can be hypothesized that the accumulation of the small inulin-type oligofructans is involved in the protection of transport tissues from oxidative damage to ensure the assimilate supply toward the endosperm. Considering that cells facilitating active transport need efficient protection of their membranes, the accumulation and possible membrane insertion of inulin-type oligofructans represents an optimal strategy to quench ^•^OH produced in direct vicinity. The flexible structure of the glycosidic linkages and sugar rings enables interaction with plasma membranes (Vereyken et al., 2003; Valluru and Van Den Ende, 2008). Accordingly, membrane stabilizing effects have been observed during abiotic stress experiments (Hincha et al., 2007; Livingston et al., 2009). Tobacco plants transformed with the 1-SST gene from *Lactuca sativa* showed a reduced electrolyte leakage when compared to the WT during cold stress treatments and thus, an increased freezing tolerance has been concluded (Li et al., 2007). Increased oligofructan levels have also been reported from cold acclimation experiments in oat (*Avena sativa*; Livingston and Henson, 1998) and were associated to membrane stabilizing effects of low DP oligofructans (Valluru et al., 2008; Livingston et al., 2009). Recently, evidence has been provided for the reaction of inulin-type oligofructans with ^•^OH radicals generated during the Fenton reaction and that the capability to scavenge ROS is more effective than for various phenolic compounds (Peshev et al., 2013). The reaction of sugars with ^•^OH leads to the formation of non-radical oxidized sugars and to degradation, recombination and regeneration products (Tudella et al., 2011; Hernandez-Marin and Martinez, 2012; Peshev et al., 2013; Peukert et al., 2014). In particular, oligofructans from barley cavity sap have been proven to react non-enzymatically with ^•^OH radicals under formation of a plethora of non-radical hexose splitting and oxidation products (Peukert et al., 2014; Matros et al., 2015). These processes might play a dual role during grain development with respect to abovementioned functions of the various soluble carbohydrates. In conclusion, the efficiency of biosynthesis, the possible interaction with biological membranes and the reactivity with ^•^OH radicals support the hypothesis that inulin-type oligofructans play an important role for maintenance of high transport activity in the developing grain (Peukert et al., 2014).

## Future Perspectives

The versatile physiological functions of oligofructans in the developing grain (summarized in **Figure 3**) have been deduced from the spatiotemporal coordination of biosynthesis and accumulation, *in vitro* experiments and results gathered from vegetative plant tissues. Future experiments will focus on grain-specific modulations of the oligofructan metabolism, with particular emphasis on the NP and endosperm tissues. Genetic approaches that alter the expression of key fructan biosynthesis genes may shed light on the influences of oligofructans on cellularization and differentiation processes within the early endosperm. Particularly, reduced 6-SFT activity in the endosperm is supposed to provide novel information about carbon partitioning, sucrose signaling, balancing of the glucose/sucrose ratio and its impact on cell proliferation/elongation. Diminishing 1-SST expression in the NP with the beginning of the storage phase would aid to elucidate if releasing of oligofructans from the maternal grain tissues into the endosperm cavity is affected. According to our model, limitations in inulin-type oligofructans could negatively affect transport processes by increased ROS inflicted damage and thereby, decrease membrane stability. In this respect, immobilized artificial membrane systems based on reconstituted liposomes (Hincha et al., 2007) might represent a useful tool to investigate the protective and membrane stabilizing effects of the various oligofructan-types under oxidative stress (e.g., ROS generation during the Fenton reaction).

The described protective function against oxidative stress in transport tissues is also supposed to be part of the processes conferring tolerance against related abiotic stresses, such as cold or drought (Kawakami et al., 2008; Keunen et al., 2013). However, studies in this field are mostly restricted to vegetative tissues during the tillering phase and not to seed development. The increasing availability of genetically characterized diverse populations and access to approaches for high-throughput phenotyping will enable detailed analysis of the correlation between cold/drought tolerance and grain oligofructan profiles in the future. Aforementioned approaches might also help to generalize deduced oligofructan-type specific functions across fructan containing plant species.

Fructans have been also described as prebiotics with health promoting effects (Roberfroid, 2007; Di Bartolomeo et al., 2013; Dwivedi et al., 2014). Especially their potential role in chemoprevention of colon carcinogenesis make these metabolites an interesting target for biomedical research (Wollowski et al., 2001; Beyer-Sehlmeyer et al., 2003; Pool-Zobel et al., 2005; Sauer et al., 2007). Recent studies indicate that inulin-type oligofructans do not only act as prebiotics via the impact on microbial composition and short chain fatty acid formation, but directly affect the colonic epithelium (Peshev and Van Den Ende, 2014) by mediating immunomodulatory effects with dedicated stimulation of Toll-like receptors (Vogt et al., 2015). Thus, cereal grains with variations in oligofructan concentrations might impact the human intestine in different ways. Such studies would open new possibilities for breeding of cereal grains that might promote human health.

We conclude that most relevant future research fields are: (i) the detailed elucidation of structure-function relationships for the various fructan-types and the variation in DP; (ii) the differentiation of oligofructan function in the various tissues and possible generalizations across the fructan accumulating plants species; and (iii) the analysis of the bioavailability, biotoxicity and bioefficacy of the various cereal oligofructan-types to carefully assign their impact on human health and enabling clear advise for future cereal breeding.

## Conflict of Interest Statement

The authors declare that the research was conducted in the absence of any commercial or financial relationships that could be construed as a potential conflict of interest.

## Footnotes

1 http://faostat3.fao.org/compare/E

2 http://apps.webofknowledge.com/
